# Antibacterial MccM as the Major Microcin in *Escherichia coli* Nissle 1917 against Pathogenic Enterobacteria

**DOI:** 10.3390/ijms241411688

**Published:** 2023-07-20

**Authors:** Yi Ma, Wei Fu, Bin Hong, Xinfeng Wang, Shoujin Jiang, Jufang Wang

**Affiliations:** 1 School of Biology and Biological Engineering, South China University of Technology, Guangzhou 510006, Chinabixinf.w@mail.scut.edu.cn (X.W.); 202021049932@mail.scut.edu.cn (S.J.); 2 Guangdong Provincial Key Laboratory of Fermentation and Enzyme Engineering, South China University of Technology, Guangzhou 510006, China

**Keywords:** *Escherichia coli* Nissle 1917, pathogenic enterobacteria, microcins, antibacterial activity, antibacterial mechanism

## Abstract

Probiotic *Escherichia coli* Nissle 1917 (EcN) possesses excellent antibacterial effects on pathogenic enterobacteria. The microcins MccM and MccH47 produced in EcN played critical roles, but they are understudied and poorly characterized, and the individual antibacterial mechanisms are still unclear. In this study, three EcN mutants (Δ*mcmA*, Δ*mchB,* and Δ*mcmA*Δ*mchB*) were constructed and compared with wild-type EcN (EcN wt) to test for inhibitory effects on the growth of *Escherichia coli* O157: H7, *Salmonella enterica* (SE), and *Salmonella typhimurium* (ST). The antibacterial effects on O157: H7 were not affected by the knockout of *mcmA* (MccM) and *mchB* (MccH47) in EcN. However, the antibacterial effect on *Salmonella* declined sharply in EcN mutants Δ*mcmA*. The overexpressed *mcmA* gene in EcN::*mcmA* showed more efficient antibacterial activity on *Salmonella* than that of EcN wt. Furthermore, the EcN::*mcmA* strain significantly reduced the abilities of adhesion and invasion of *Salmonella* to intestinal epithelial cells, decreasing the invasion ability of ST by 56.31% (62.57 times more than that of EcN wt) while reducing the adhesion ability of ST by 50.14% (2.41 times more than that of EcN wt). In addition, the supernatant of EcN::*mcmA* culture significantly decreased the mRNA expression and secretion of IL-1β, TNF-α, and IL-6 on macrophages induced by LPS. The EcN::*mcmA* strain generated twice as much orange halo as EcN wt by CAS agar diffusion assay by producing more siderophores. MccM was more closely related to the activity of EcN against *Salmonella,* and MccM-overproducing EcN inhibited *Salmonella* growth by producing more siderophores-MccM to compete for iron, which was critical to pathogen growth. Based on the above, EcN::*mcmA* can be developed as engineered probiotics to fight against pathogenic enterobacteria colonization in the gut.

## 1. Introduction

Pathogenic enterobacteria and related diseases have attracted increasing attention from consumers and food safety regulators because of their undeniable impact on health and the economy [1]. Enterohaemorrhagic *Escherichia coli* (EHEC) O157: H7 and *Salmonella* are major pathogenic enterobacteria that cause potentially fatal diseases such as hemorrhagic colitis, acute gastroenteritis, and salmonellosis [2]. Shiga toxin 1 (Stx1) and 2 (Stx2) are among the virulence factors that contribute to the pathogenesis of EHEC [3]. *Salmonella enterica* (SE) and *Salmonella Typhimurium* (ST) accounted for the majority of *Salmonella* and were considered to be responsible for most of the salmonellosis infections [4]. *Salmonella* can even thrive in the inflamed gut using unique carbon and energy [5] and have a partial nutritional immune response to the antibacterial proteins secreted by the host [6]. The pathogenesis and colonization of host tissues by *Salmonella* depend on special systems called secretory systems type III (T3SS), which contain SPI1 and SPI2, which play different roles in infection [7]. The SPI1 system was associated with diarrhea and intestinal colonization. In contrast, SPI2 T3SS plays a key role in the survival of *Salmonella* in macrophages [8]. Broad-spectrum antibiotics are basically unsuccessful in the treatment of intestinal bacterial infections.

Probiotics have been found to have positive effects through a variety of mechanisms, such as reducing pathogen adhesion and invasion caused by competitive rejection, anti-inflammatory activity, nutritional competition, bacteriocin production, and reducing host cell apoptosis [9]. *Escherichia coli* Nissle 1917 (EcN) [10], a widely used probiotic strain, was isolated from Alfred Nissle, a soldier who was resistant to severe diarrhea outbreaks during the First World War [11]. The good probiotic activity of EcN in humans and animals has been reported in several studies [12,13], and EcN has been proven to be very effective in relieving ulcerative colitis [14]. A recent in vitro study showed that EcN inhibits EHEC growth and adhesion, and in vivo study indicated that it protected mice from EHEC, including EHEC O157:H7 [15]. As of yet, no specific treatment has been approved for EHEC infection, and antibiotics may increase Shiga toxin production [16]. Additionally, it was reported that EcN can reduce the adhesion and invasion of several intestinal pathogens into mammalian cells, including *Yersinia*, *Salmonella*, *Shigella,* and atypical intestinal pathogenic *Escherichia coli* [17].

The antibacterial activity of EcN is associated with the production of two microcins (Mccs), M (MccM) and H47 (MccH47). Mccs are peptides synthesized from ribosomes and modified after translation that have effective antibacterial activity [18,19]. Some symbionts and pathogens synthesize and secrete siderophores to obtain iron under iron deficiency [20,21]. Siderophores are small-molecular-weight substances that can specifically bind iron and provide it to microbial cells [22]. Iron, as a cofactor in biochemical metabolic reactions, is a fundamental element of microbial growth and plays a key role in the life activities of microorganisms, such as electron transfer [23]. Microorganisms can secrete siderophores to chelate iron to satisfy self-growth needs in iron-deficient environments [24]. Because of the large number of siderophores and a variety of siderophores receptors, the EcN competes for iron to reduce ST intestinal colonization [25]. Moreover, MccM and MccH47 are known as “siderophore-Mcc” because they are modified at the C-terminus by linking linearized and glycosylated derivatives of the catechol siderophore enterobactin [26]. The catechin-siderophore receptor of the targeted bacteria is recognized by siderophores [27]. Then, siderophore-Mcc imitates the iron-siderophore complex by entering and killing sensitive bacteria through a “Trojan horse” strategy [28]. But the antibacterial mechanism of MccM or MccH47 on pathogenic enterobacteria was rarely reported.

Our study aimed to verify whether the microcins generated by wild-type EcN (EcN wt) exerted an antibacterial role. We constructed three EcN mutants (Δ*mcmA*, Δ*mchB,* and Δ*mcmA* Δ*mchB*) by the CRISPR-Cas9 method to eliminate the precursor genes encoding two microcins, MccM and MccH47. We confirmed that MccM was more important for the antibacterial effects of EcN wt, and then MccM was successfully overexpressed in EcN. At last, the antibacterial effects of EcN::*mcmA* were compared with those of EcN wt, including the reduction in growth, adhesion, and invasion of *Salmonella* and the inhibition of macrophage inflammatory cytokine expression.

## 2. Result

### 2.1. Effect of Probiotic EcN on EHEC O157: H7 Growth and Stx Gene Expression

**Figure 1:**
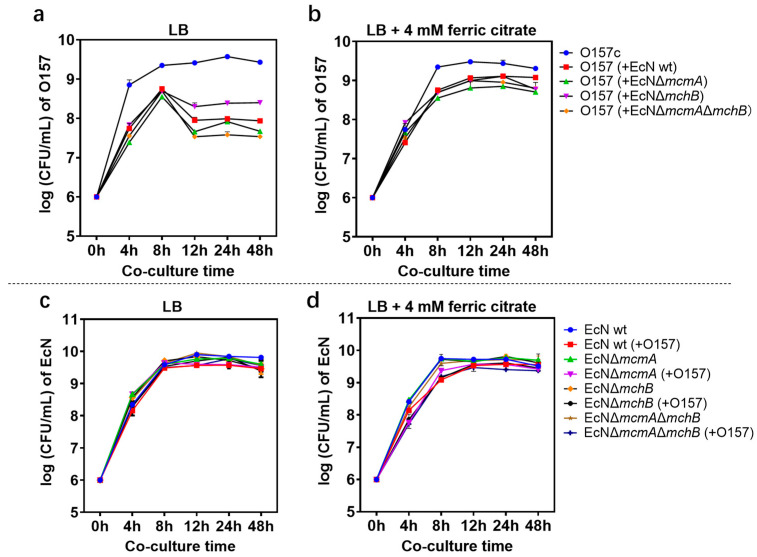
The activity of EcN microcins against EHEC O157: H7. EHEC O157: H7 CFU mL^−1^ in vitro when grown alone or in antagonism with EcN wt or mutants in (**a**) LB medium or in (**b**) iron-rich medium (LB supplemented with 4 mM iron citrate). EcN wt or mutants CFU mL^−1^ when grown alone or in antagonism with EHEC O157:H7 in (**c**) LB medium or in (**d**) iron-rich medium.

**Figure 2:**
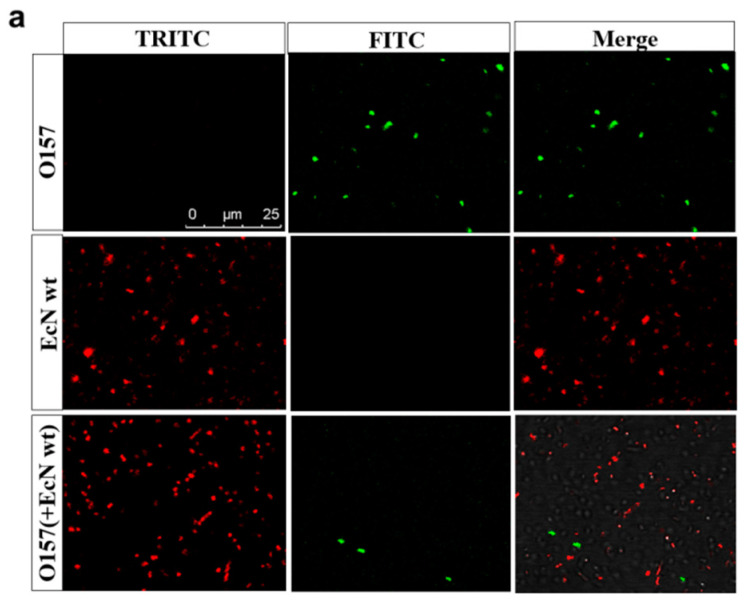

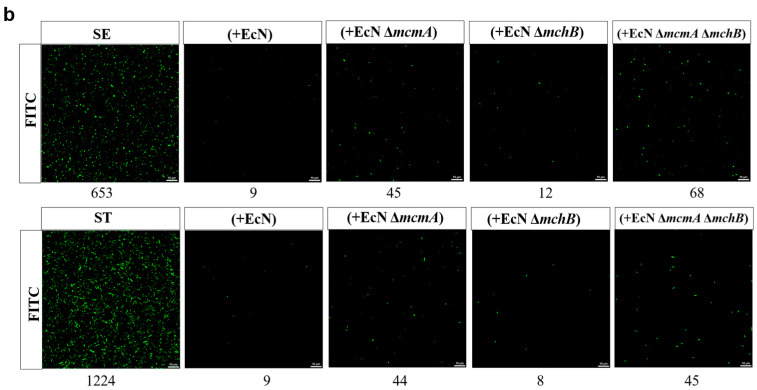
Spectrofluorometric assays. (**a**) EcN wt and EHEC O157: H7 express the fluorescent proteins mCherry and GFP, respectively. After 4 h of co-culture in LB medium, the survival number of bacteria was characterized by fluorescence number. Scale bars, 25 μm. (**b**) Fluorescence produced by GFP of SE/ST when grown alone or in antagonism with EcN wt or mutants in iron-limited conditions for 24 h. Cells were counted (under the picture) using the software ImageJ v1.8.0. Scale bars, 10 μm.

The bacterial concentrations of EHEC O157: H7 monocultures as well as co-cultures with EcN wt or mutants were determined at the beginning of the experiment (0 h) and at 4 h, 8 h, 12 h, and 24 h. In LB co-culture experiments, the growth of EHEC O157: H7 was significantly inhibited by EcN after 8 h. The EcN Δ*mcmA*Δ*mchB* strain had the best inhibition effect, reaching 99% in 24 h compared with monoculture as a control; there was no significant difference from EcN wt (Figure 1a). Of course, the number of EHEC O157: H7 inhibited by EcN wt was reduced by 92% at 4 h. In the fluorescence microscope image, it could be seen that the green fluorescence was significantly less than the red fluorescence (Figure 2a). Under iron-rich conditions, the growth inhibition of EcN on EHEC O157: H7 was weakened, indicating that ions had an impact on the antibacterial activity of EcN (Figure 1b). Unexpectedly, no matter whether in iron-limited or iron-rich conditions, the growth of EcN was not influenced by EHEC O157: H7 (Figure 1c,d). The expression of *stx* mRNA was detected after 2 h, 4 h, and 24 h co-culture of EHEC O157: H7 with EcN (Supplementary Figure S3). RT-PCR data showed that *stx* expression of EHEC O157: H7 incubated with EcN was significantly reduced compared with EHEC O157: H7 alone in LB medium or iron-rich medium. The strain EcN Δ*mcmA* Δ*mchB* showed the best effect.

### 2.2. E. coli Nissle 1917 Significantly Reduced the Growth of Salmonella in Iron-Limited Media

**Figure 3:**
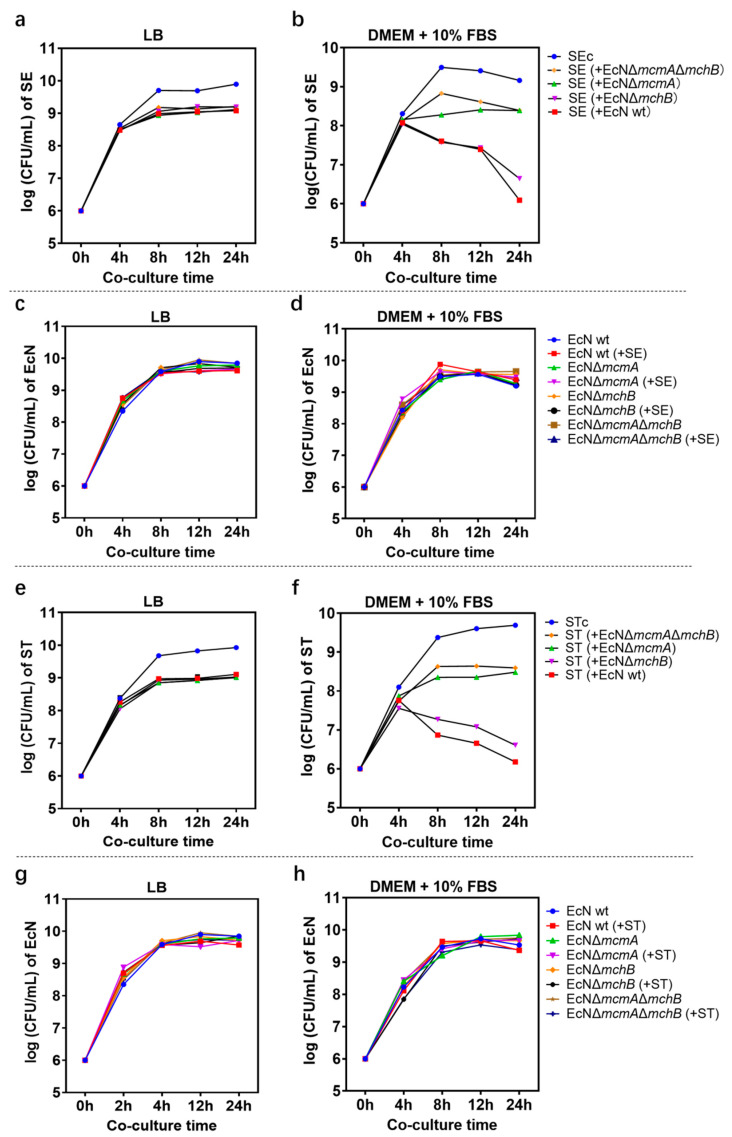
The activity of EcN microcins against *Salmonella* in vitro. SE/ST CFU mL^−1^ when grown alone or in antagonism with EcN wt or mutants in (**a**,**e**) LB medium or in (**b**,**f**) iron-limited conditions. EcN wt or mutants CFU mL^−1^ when grown alone or in antagonism with SE/ST in (**c**,**g**) LB medium or in (**d**,**h**) iron-limited conditions.

Growth inhibition of *Salmonella* by EcN increased over time after 4 h of co-culture (Figure 3). The antagonistic effect of EcN on *Salmonella* in LB medium is equivalent to that in iron-rich medium supplemented with 4 mM iron citrate, so LB medium was regarded as an iron-rich condition. Under the iron-limiting condition, compared with SE monoculture as a control, the number of SE co-cultured with EcN wt decreased by 99% (~100-fold) in 12 h and 99.9% (~1000-fold) in 24 h, whereas co-cultured with EcN Δ*mchB* decreased by 98.7% (~100-fold) in 12 h and 99.2% (~100-fold) in 24 h (Figure 3a,b). Similarly, the number of ST was significantly reduced at 8 h, and the EcN wt strain had the best inhibitory effect: 99.69% at 8 h, 99.88% (~1000-fold) at 12 h, and 99.97% at 24 h. When co-cultured with EcN Δ*mchB*, the number of ST was suppressed at 99.22% in 8 h, 99.70% in 12 h, and 99.92% (~1000-fold) in 24 h (Figure 3e,f). Similar results were found in fluorescence microscope images (Figure 2b), where the green fluorescence number decreased sharply when EcN wt and EcN ΔmchB were added to co-culture compared with the control (Supplementary Figure S4).

On *Salmonella*, the best effect was reached at 24 h. In addition, the growth inhibitory effect of the mutant strain EcN Δ*mcmA* on *Salmonella* was similar to that of EcN Δ*mcmA*Δ*mchB* and significantly lower than that of EcN wt (Figure 3b,f). The results indicated that MccM influenced SE/ST growth more effectively under iron-deficiency stress. However, when four EcN strains were co-cultured with *Salmonella*, the number of EcN strains was unaffected compared to EcN alone (Figure 3c,d,g,h).

### 2.3. The Engineered Strain EcN::mcmA Significantly Reduced the Growth of Salmonella in Iron-Limited Media

**Figure 4:**
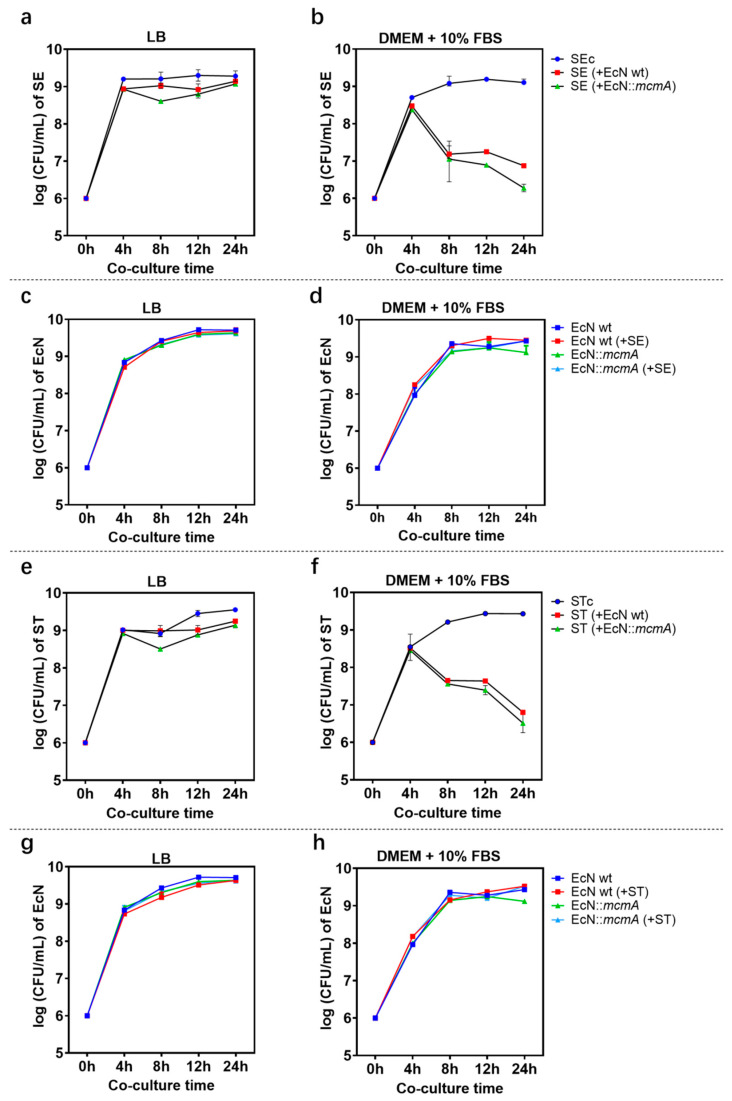
The activity of EcN wt and EcN::*mcmA* against *Salmonella* in vitro. SE/ST CFU mL^−1^ when grown alone or in antagonism with EcN wt and EcN::*mcmA* in LB medium (**a**,**e**) or in iron-limited conditions (**b**,**f**). EcN wt and EcN::*mcmA* CFU mL^−1^ when grown alone or in antagonism with SE/ST in (**c**,**g**) LB medium or in (**d**,**h**) iron-limited conditions.

Antibacterial experiments in vitro showed that engineered strains EcN::*mcmA* and EcN wt could inhibit the growth of *Salmonella* in LB medium or in iron-limiting conditions, and the inhibition effect was particularly significant in an iron-limited environment. Among them, the antibacterial effect of the engineered strain on *Salmonella* was stronger than that of EcN wt (Figure 4). In LB medium, the engineered strain reached its maximum effect at 8 h, reducing the number of SE by 75.8% and ST by 61.7%. However, the inhibition rate of EcN wt on SE was 36.4%, and there was no effect on ST (Figure 4a,e). Under iron-limiting conditions, EcN::*mcmA* had 0.8 log and 0.23 log more reductions in SE and ST than EcN wt, respectively, in 24 h (Figure 4b,f,i). These results indicated that microcins produced by probiotic EcN had very significant antibacterial activity against *Salmonella,* and engineered strains were more effective. Similarly, *Salmonella* did not affect the growth of EcN, and the growth curve of the engineered strain was similar to EcN wt, so overexpression of MccM would not increase the burden on EcN (Figure 4c,d,g,h).

**Figure 5:**
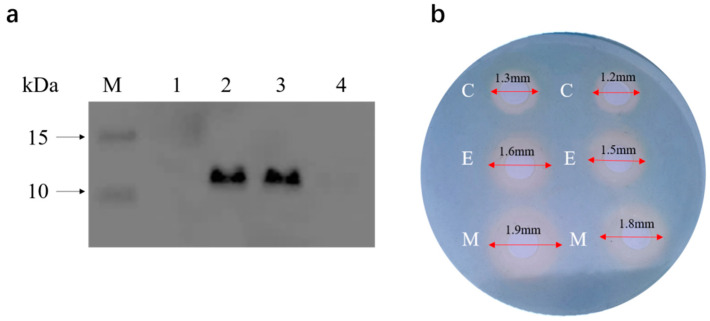
(**a**) Western blot analysis of EcN cells expressing microcin M protein was performed. Lane 1, whole-cell lysate of EcN wt; lane 2, whole-cell lysate of EcN wt harboring pUC19-*mcmA*; lane 3, supernatant of EcN wt harboring pUC19-*mcmA*; lane 4, precipitate of EcN wt harboring pUC19-*mcmA*; lane M, protein marker 26616. (**b**) CAS agar diffusion (CASAD) assay. The supernatant of EcN wt (E) and engineered strain EcN::*mcmA* (M) cultured overnight in iron-limited conditions was detected for the generation of siderophores using a modified CAS agar diffusion method. Culture medium as negative control (C). The orange halo that formed around each pore indicated the presence of siderophores in the supernatant.

After recombinant plasmids were transformed into EcN wt, the whole cell lysates of recombinant EcN were detected by SDS-PAGE for confirmation of MccM overexpression. The recombinant protein MccM could hardly be detected by Coomassie blue staining due to its low expression. Therefore, Western blot (WB) analysis was carried out using HRP-conjugated 6*His antibody for the immunodetection of the recombinant protein. The molecular weight of MccM was 11.4 kDa, calculated from the amino acid sequence. The bands of WB results corresponding to the 11.4 kDa protein were detected in whole-cell lysates of the recombinant EcN harboring pUC19-*mcmA*, suggesting that MccM was successfully expressed (Figure 5a).

### 2.4. Antibacterial Mechanism of the Engineered Strain EcN::mcmA

**Figure 6:**
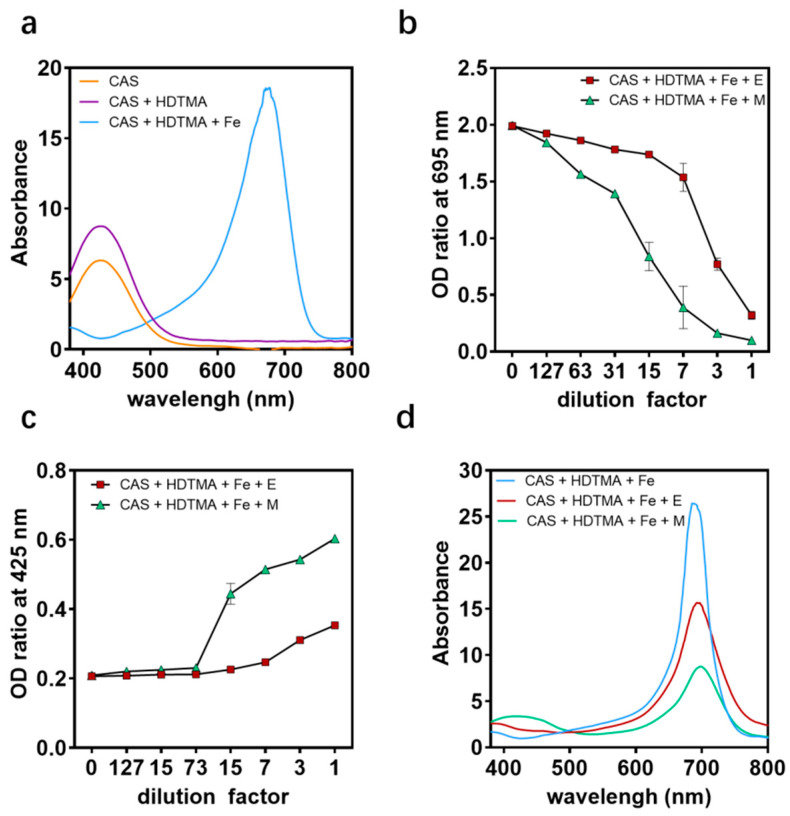
Determination of various components in the UV visible absorption spectrum. (**a**) CAS, CAS+Fe, and CAS+HDTMA complexes all have absorption peaks near 425 nm. When constructing a ternary detection complex CAS+Fe+HDTMA, the absorption peak shifted to 695 nm. (**b**–**d**) When siderophores chelate iron, the peak at 695 nm linearly decreases, and the peak at 425 nm increases. The supernatant of the EcN wt (E) and engineered strain EcN::*mcmA* (M) cultured for 24 h were added to CAS+Fe+ HDTMA according to the dilution times, and the absorbance was measured at 695 nm and 425 nm, respectively (**b**,**c**). The absorbance of the strains’ supernatant diluted 7 times was measured (**d**).

At present, the detection method commonly used for siderophores is the general CAS agar plate method. When the siderophores produced by microorganisms combine with the blue complex composed of CAS, cetyltrimethylammonium bromide (HDTMA), and iron, it will take away the iron and change color from blue to orange, producing a distinct siderophore halo. The CAS agar diffusion assay found that the engineered strain EcN::*mcmA* could produce an orange halo of 0.6 mm, which was twice the orange halo (0.3 mm) produced by EcN wt (Figure 5b). It could be seen from the CAS liquid measurement that the absorbance at 695 nm decreased with the increase in the amount of supernatant added to the bacterial liquid, and the absorbance at 425 nm increased. When diluted 7-fold, the decrease in absorbance at 695 nm of the engineered strain EcN::*mcmA* was 1.7 times that of the EcN wt (Figure 6a–d), which indicated that the engineered strain produced more siderophores than EcN wt.

### 2.5. The Engineered Strain EcN::mcmA Significantly Inhibited the Adhesion and Invasion of Salmonella to Intestinal Epithelial Cells

**Figure 7:**
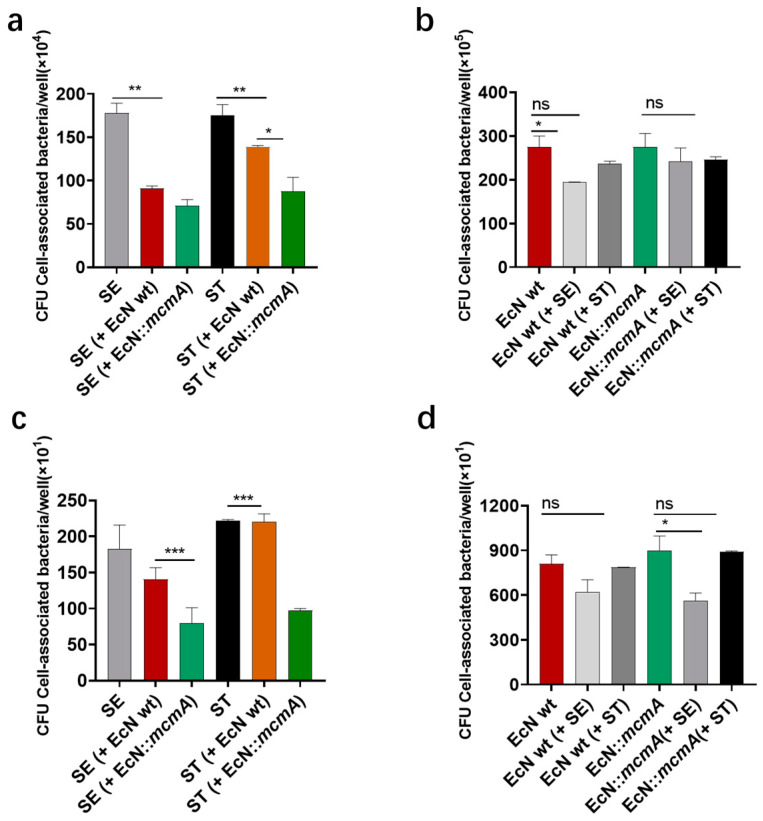
Adhesion and invasion of *Salmonella* (**a**,**c**) and EcN strains (**b**,**d**) to intestinal epithelial cells HT29. EcN and *Salmonella* were co-infected at the same infection rate (MOI = 100), with separate infection as the control. Infections were performed for 1 h and 3 h. The number of bacterial adhesion and invasion of HT29 cells was counted by plate counting. (“ns”, *p* > 0.05; “*”, *p* < 0.05; “**”, *p* < 0.01; “***”, *p* < 0.001). Data are given as the mean ± S.E.M. of three to five separate experiments.

After the co-infection of engineered strain EcN::*mcmA* and *Salmonella* with intestinal epithelial cells HT-29, compared with *Salmonella* infection alone, the adhesion number of SE and ST decreased by 60.01% and 50.14%, respectively, 1.23 times and 2.41 times of the co-infection with EcN wt (Figure 7a). Similarly, the invasion number of SE and ST decreased by 56.16% and 56.31%, respectively, 2.44 times and 62.57 times the co-infection with the wild-type EcN strain (Figure 7c). However, *Salmonella* had little impact on the adhesion and invasion of EcN strains (Figure 7b,d). The above results indicated that the EcN strain, after overexpressing the *mcmA* gene, had an enhanced inhibitory effect on the adhesion and invasion of *Salmonella* into HT-29 cells.

### 2.6. The Engineered Strain EcN::mcmA Significantly Inhibited the Expression of Inflammatory Cytokines

**Figure 8:**
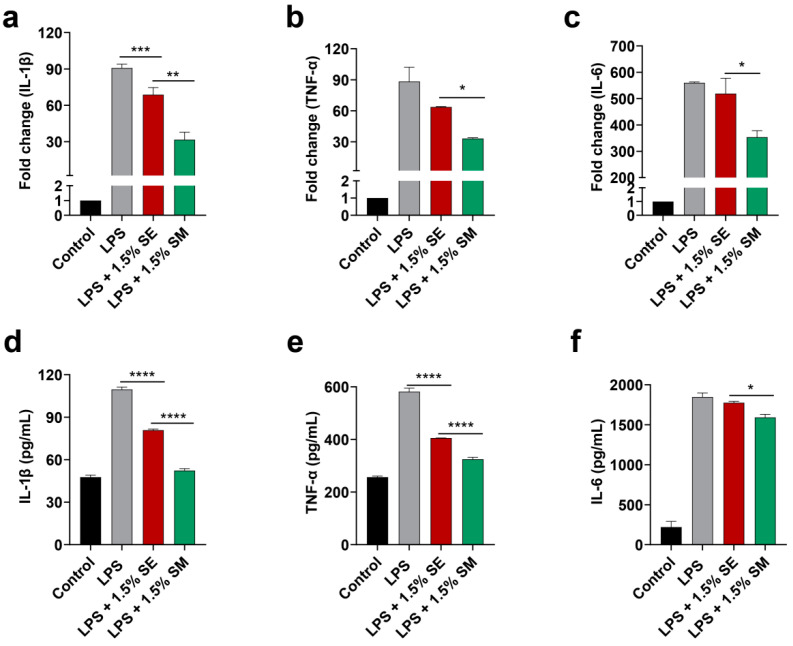
Engineered strain EcN::*mcmA* inhibited the expression of inflammatory cytokines. The 1.5% supernatant of EcN wt (1.5% SE) and EcN::*mcmA* (1.5% SM) inhibited the mRNA expression (**a**–**c**) and the secretion (**d**–**f**) of IL-1β, TNF-α, and IL-6 by RAW264.7 cells infected with LPS. (“*”, *p* < 0.05; “**”, *p* < 0.01; “***”, *p* < 0.001; “****”, *p* < 0.0001).

To investigate the anti-inflammatory effect of the engineered strain EcN::*mcmA*, RT-PCR and ELISA were used to detect the expression of TNF-α, IL-1β, and IL-6 after the treatment of CT26-mediated RAW264.7 cells with the supernatant of EcN wt and EcN::*mcmA*. The results indicated that the mRNA expression of IL-1β and secretion of TNF-α and IL-1β induced by LPS were significantly inhibited after the 1.5% supernatant of EcN wt treatment (*p* < 0.05, Figure 8a,d,e), while the effects of it on the expression of IL-6 were not significantly altered (*p* > 0.05, Figure 8c,f). Moreover, compared with EcN wt, engineered strain EcN::*mcmA* has a more obvious inhibitory effect on the expression of inflammatory cytokines, and 1.5% supernatant significantly reduces the mRNA expression as well as the secretion of TNF-α, IL-1β, and IL-6 produced via the induction of macrophages by LPS (*p* < 0.05, Figure 8a–f).

## 3. Discussion

The overuse and misuse of antibiotics, coupled with the lack of new antibiotics, induced the emergence and spread of multi-resistant bacteria [29]. This phenomenon constitutes an enormous risk of global morbidity and mortality [30]. Microcins appeared to be a potential antimicrobial alternative to conventional antibiotics. The main challenge in using Mccs was delivering them to the infection site in sufficient quantities [31]. Thus, engineered probiotics were considered in situ producers of Mccs to combat pathogenic enterobacteria or reduce the colonization of multidrug-resistant bacteria [32]. EcN has been used as a probiotic for more than 100 years, and several different health advantages have been reported [33]. Therefore, we constructed an engineered EcN strain as an in situ producer, producing a large amount of MccM to antagonize pathogenic enterobacteria and provide the possibility for new antibacterial agents.

The EHEC pathogen is most commonly transmitted through contaminated food and water, and the Shiga toxin contributes to the pathogenicity of the organism [34]. Our research found that probiotic EcN reduces the growth and Shiga toxin expression of EHEC O157: H7, which was consistent with previous studies, and that the growth of EcN was not affected by EHEC O157: H7. However, this specific inhibition mechanism has not been studied. The EcN Δ*mcmA* Δ*mchB* strain had the best inhibition effect, reaching 99% in 24 h, indicating that microcins are not associated with this inhibitory effect in our study. This was consistent with previous research [35]. Some studies show that EcN may inhibit the expression of adhesin in EHEC or induce the expression of human β-defensin 2 through the flagellum to kill EHEC [36]. In our experiment, we found that EcN more significantly inhibited the growth of EHEC O157: H7 in iron-limiting conditions. EcN has a competitive advantage in the uptake of iron in cases of iron deficiency. There are some proteins connected with the iron uptake system that can bring advantages to EcN in outgrowing and competing with pathogens using similar siderophores [37]. Only a few studies have researched the role of microcins on the Shiga toxin production of EHEC O157: H7. According to our study, Shiga toxin gene expression was downregulated in the absence of microcins, which suggested that microcins might play a role in promoting Shiga toxin production. One study found that microcins of EcN increased Shiga toxin production through an SOS-dependent pathway, similar to how ciprofloxacin produces Shiga toxin [38]. So, the mechanism of EcN’s effects on the growth of EHEC O157: H7 needs to be investigated in the future.

EcN microcins were shown to have antibacterial activity against *Salmonella* in iron-limited environments [27]. *Salmonella* was the main cause of acute gastroenteritis, which was characterized by inflammatory diarrhea. Some mechanisms were utilized by the host to limit the availability of iron during inflammation [39,40]. When *Salmonella* was co-cultured with EcN wt, the number of *Salmonella* decreased significantly in iron-limiting (~1000-fold) and less in iron-rich (~7-fold) conditions at our experiments, which was probably because of microcins, and siderophores were only expressed when iron deficiency occurred [25]. Interestingly, the growth of four EcN was not affected by *Salmonella*. Research showed that the single deletion of MccM structural gene *mcmA* or MccH47 structural gene *mchB* did not weaken the inhibitory effect of EcN on *Escherichia coli* LF82 but weakened it after the combined deletion of *mcmA* and *mchB* [41]. In contrast, in our study, the antibacterial activity of EcN against *Salmonella* was affected by a single deletion of the MccM structural gene *mcmA*. Therefore, we deduced that MccM was more important than MccH47 in EcN against *Salmonella*.

Next, MccM was successfully overexpressed in EcN in our study. We showed that overexpression of the precursor of MccM in the engineered probiotic bacteria EcN::*mcmA* had a more effective antibacterial effect against *Salmonella* and produced more siderophores compared with EcN wt. It is a common strategy for pathogens to survive in iron-limited environments where they produce siderophores, which are small, potent iron-chelating molecules. Similarly, EcN produces a variety of siderophores, which makes it superior in competing with other pathogenic enterobacteria for iron, thus promoting its colonization in the intestine. A recent study demonstrated that multiple siderophores enable EcN to outcompete *S. Typhimurium* for iron acquisition [25]. Therefore, overexpression of MccM in our study may promote siderophores production, which can improve the competitive advantage of iron to outcompete *Salmonella*. Enterobactin is a catechol-type siderophore that is used to modify microcins. In the MccH47 genetic system carried by strains, *mchS4* was identified as the single gene responsible for enterobactin overproduction. The MccH47 genetic system promotes enterobactin production, and the enterobactin synthesis pathway plays a role in MccH47 synthesis [42]. MccH47 was widely heterologously expressed in EcN or other *Escherichia coli* strains [43]. Due to the post-translational modification of the enterobactin moiety [44], MccH47 enters the pathogenic bacteria via the catecholate siderophore receptors (FepA, Fiu, or Cir), which are then applied to the F0 proton channel, triggering the unregulated entry of protons and thereby dissipating the membrane potential. The mechanism of MccM is not clear, but it may be consistent with MccH47 [45]. Therefore, the future of research should focus on MccM, including antibacterial mechanisms, the construction of engineered bacteria, heterologous expression, etc.

As *Salmonella* colonization and pathogenicity depend on adhesion to the intestinal epithelium, inhibiting its adhesion ability can prevent bacterial invasion and reduce intestinal colonization. The bacterial adhesion to intestinal epithelial cells has long been considered an ideal prerequisite for probiotic strains [46]. Therefore, a lot of probiotics have the ability to adhere to the intestine [47]. It has been demonstrated in our study that the engineered strain EcN::*mcmA* prevents *Salmonella* from colonizing intestinal epithelial cells. The repression on adhesion and invasion of SE and ST was higher than that of EcN wt, respectively. EcN has been proven to possess anti-inflammatory properties in several cell types, including human colonic epithelial cells [48]. It has been suggested that intestinal infections may be triggered by lipopolysaccharides (LPS) released by Gram-negative bacteria such as *Salmonella* [49]. Macrophages play a dual role in the process of *Salmonella* invading the host. In addition to their role in the immune response, macrophages are also host cells for *Salmonella*, facilitating its systemic spread and causing infection [50]. In this study, we found that the engineered strain EcN::*mcmA* significantly inhibited the LPS-induced expression of IL-1β, TNF-α, and IL-6 in RAW264.7 macrophages compared with EcN wt.

## 4. Materials and Methods

### 4.1. Strains, Plasmids and Culture Conditions

*E. coli* Nissle 1917 (EcN; Mutaflor), Enterohemorrhagic *E. coli* (EHEC) O157:H7, the pathogen *S. enterica* (SE), and the *S. Typhimurium* (ST) used were listed in Table 1. Plasmid pUC19 with chloramphenicol or ampicillin resistance was electroporated into probiotic EcN and pathogenic enterobacteria for screening. The plasmids and partial primers used in this research are listed in Table 1. All strains were cultured in LB broth or agar plates with antibiotics at 37 °C. The antibiotic concentrations are chloramphenicol (Cm^+^, 25 µg/mL), spectinomycin (Sm^+^, 25 µg/mL), ampicillin (Amp^+^, 100 µg/mL), or kanamycin (Kan^+^, 50 µg/mL).

### 4.2. Generation of Bacterial Mutants

Mutants in EcN (EcN Δ*mcmA*, EcN Δ*mchB*, and EcN Δ*mcmA* Δ*mchB*) were constructed using CRISPR/Cas9 gene targeting. The gene-specific single guide RNAs (sgRNA) were designed using the software sgRNAcas9_3.0.5 (http://biootools.com/col.jsp?id=140) on 12 June 2021. The program was written in Perl (http://www.perl.org). The DNA sequences of the target genes *mcmA* (UniProtKB-Q2WEK3) and *mchB* (UniProtKB-P62530) were found on the EcN genome (GenBank: CP022686.1), and the sgRNA primers A20-F/A20-R and B20-F/B20-R (respective upper case N20 sequence of 20 bp) were designed using the procedures mentioned in this method. The selected homology arms H1 and H2 were about 500 bp upstream and downstream of the target genes *mcmA* and *mchB*, and the designed amplification primers were AH1-F and AH1-R, AH2-F and AH2-R, BH1-F and BH1-R, and BH2-F and BH2-R. The strain names of the mutants and primers are listed in Table 1. Construction of sgRNA recombinant plasmids by double digestion, restriction-free cloning technology (RF cloning) [51], and seamless cloning [52]. Subsequently, the primers Δ *mcmA*-F/Δ *mcmA*-R and Δ *mchB*-F/Δ *mchB*-R were used for knockout verification and sent to the company (Sangon Biotech, Shanghai, China) for sequencing. Subsequently, the plasmid pUC19-mCherry containing chloramphenicol resistance and the plasmid pUC19-sGFP containing ampicillin resistance were introduced into the constructed EcN and *Salmonella* for resistance screening.

### 4.3. In Vitro Growth Assays

EcN microcins were tested in vitro against EHEC O157: H7, SE, and ST under iron-limited and iron-rich conditions. Strains were first cultured overnight at 37 °C in the resistant LB added with 0.2 mM 2,2′-dipyridyl (Sangon Biotech, Shanghai, China) (Supplementary Figure S2a) aerobically. EcN and pathogenic enterobacteria were inoculated in a 1: 1 ratio. Approximately 1 × 10^6^ CFU mL^−1^ of overnight culture (calculated according to the formula of the growth curve, Supplementary Figure S1) was inoculated into 10 mL of iron-limited conditions (DMEM with 10% fetal bovine serum (FBS); Procell, Wuhan, China) and iron-rich medium (LB supplemented with 4 mM iron citrate; Sigma, Darmstadt, Germany) (Supplementary Figure S2b), previously described [25]. Four EcN strains (EcN wt, EcN Δ*mcmA*, EcN Δ*mchB*, and EcN Δ*mcmA* Δ*mchB*) were respectively inoculated in competition with pathogenic enterobacteria (EHEC O157: H7, SE, and ST). At 0, 4, 8, 12, and 24 h after inoculation, serial dilutions of each strain were plated to determine the strain counts. In addition, four EcN strains and pathogens were screened with chloramphenicol and ampicillin-resistant plates, respectively.

### 4.4. Quantitative Real-Time PCR

Bacterial co-cultured liquid with four EcN strains and EHEC O157: H7 for 24 h was collected for analysis of *stx* gene mRNA expression by real-time PCR (RT-PCR), and total RNA was extracted from the bacterial cells with a total RNA extraction kit (Tiangen, Beijing, China). All RNA samples were reverse transcribed using reagents (Novizan, Nanjing, China). RT-PCR was performed with SYBR Green (Yisheng, Shanghai, China) and a Roche LightCycler^®^ 96 instrumentation system (Roche, Basel, Switzerland). Analyses were conducted using the comparative 2^∆∆Ct^ method.

### 4.5. Spectrofluorometric Assays

In this experiment, the pUC19 plasmid was used as the expression vector for red fluorescent protein (mCherry) and green fluorescent protein (GFP). Strains were inoculated in an iron-limited medium or an iron-rich medium, as mentioned above. Then the cultured cells were centrifuged at 4000× *g* for 15 mins. The cells were collected, and the supernatant was discarded. After washing with sterile water three times, 200 μL of resuspension was placed on a 96-well glass bottom plate (a special culture plate for laser confocal) with a specification of 1.5 high-level glass slides (thickness: 0.17 ± 0.005 mm, Cellvis) and observed by a confocal laser scanning microscope (Leica, Wetzlar, Germany). The excitation wavelengths were 570 and 480 nm, respectively.

### 4.6. Protein Overexpression and Western Blot Analysis

The overexpression vector pUC19-*mcmA* was constructed by seamless cloning, and then MccM was overexpressed in EcN. Using pUC-*mcmA*-F/pUC-*mcmA*-R and pUC19-F/pUC19-R as primers (Table 1), the *mcmA* gene fragment was amplified from the EcN genome, and the linearized pUC19 plasmid was from the pUC19 plasmid with the chloramphenicol resistance gene, respectively. Then, they were connected by ligase 2 × Seamless Master Mix (Sangon Biotech, China) and electrically transferred to EcN to construct the engineered strain EcN::*mcmA.* The strains were cultured overnight in a selective medium containing 25 µg/mL chloramphenicol, then diluted at 1: 50 and cultured at 37 °C (220 rpm) for 4–8 h to fully express the protein. Overexpression of microcin M was determined by Western blotting [53].

### 4.7. CAS Agar Diffusion Assay (CASDA)

Due to the chelation effect of siderophores on iron, chromium azurol S (CAS) agar analysis detected that the color of the CAS–iron complex changed from blue to orange. The CAS agar formula used in this experiment was modified [54]. Firstly, 60.5 mg of CAS solution was dissolved in 50 mL of water, and then the deionized water was mixed with 10 mL of iron III solutions. With stirring, the solution was mixed slowly with 72.9 mg of detergent (HDTMA) in 40 mL of water. Afterward, the obtained dark blue solution was mixed with 900 mL of water, including 9000 mg agar, 295.25 mg NaH_2_PO_4_·2H_2_O, 1213.5 mg Na_2_HPO_4_·12H_2_O, 125 mg NH_4_Cl, 37.5 mg KH_2_PO_4_, and 62.5 mg NaCl, pH (6.8). Subsequently, holes with a diameter of 6 mm were punched and sealed on the improved CAS agar plate and then stored at 4 °C. Every hole was filled with 100 μL of iron-limited medium and the supernatant of EcN wt or the engineered strain EcN::*mcmA*. The test strains were grown in iron-limited conditions at 37 °C and 220 rpm for 24 h. After centrifugation at 4 °C for 10 min at 4000× *g*, the culture supernatant was collected and filtered for disinfection (pore size 0.22 µm) and then analyzed for siderophores production with the previously described improved CAS agar diffusion method.

### 4.8. CAS Assay Methods

CAS colorimetric analysis was used to assess iron removal from a CAS–HDTMA–Fe ternary complex. This blue complex was composed of organic dyes, metals, and surfactants and had an absorption peak at 695 nm. When siderophores replaced iron in the analytical complex, the color changed to yellow, and the absorption peak shifted to 425 nm. The hexadentate siderophores removed iron from the analytical complex in a 1:1 ratio, so the reduced absorption at 695 nm due to iron removal was proportional to the siderophores concentration in the sample. The detailed operation was described in a previous study [54]. The supernatant of EcN wt or engineered strain EcN::*mcmA* was added in diluted proportions based on CAS+HDTMA+Fe.

### 4.9. Adhesion and Invasion Inhibition Assays

HT29 cells were seeded on a 24-well cell culture plate (JET, Guangzhou, China), with 4 × 10^5^ cells per well, cultured for 20 h, and the cell monolayers were washed three times with phosphate-buffered saline (PBS, pH = 7.2). Every single molecular layer was infected with multiple infections (MOI) of 100 bacteria per epithelial cell in 1 mL of cell culture DMEM.

After infection at 37 °C, 5% CO_2_ for 1 h, the infected cell monolayers were washed with PBS three times. Cells were lysed in deionized water using 1% Triton X-100 (Sigma), which does not affect the vitality of bacteria for at least 30 mins. To determine invasiveness, fresh DMEM containing 1% penicillin and streptomycin was added to kill the bacteria on the surface of the cells and determine the bacteria in the cells after incubating for 2 h. Subsequently, the bacteria were counted using resistant agar plates. Repeat three to five experiments for each measurement.

### 4.10. Anti-Inflammatory Activity of E. coli Nissle 1917

*E. coli* Nissle1917 was cultured overnight at 37 °C, and then the overnight culture of 1 × 10^6^ CFU mL^−1^ was inoculated in 10 mL of iron-limited conditions for 24 h at 37 °C. The culture was then centrifuged at 10,000× *g* for 10 min at 4 °C to create a supernatant, and then Tris-HCL was used to adjust the pH value to 7.2~7.4. Finally, they were filtered through 0.22 µm filters.

The CT26 and RAW246.7 were cultured in DMEM medium with 10% FBS and 1% penicillin and streptomycin at 37 °C in 5% CO_2_. For transwell experiments, CT26 cells (1 × 10^4^ cells/well) in the logarithmic growth phase were inoculated in the inserts of 24-well plates in a transwell system (1 µM nucleopore size; Costar, Corning Inc, New York, NY, USA) and placed above the 600 μL medium for two days. For determining proinflammatory cytokine production, RAW246.7 cells (5 × 10^4^ cells/well) were seeded into 24-well cell culture plates (JET, Guangzhou, China) and cultured at 37 °C until confluence was reached. Monolayers of RAW246.7 cells were initially stimulated for 20 h with 1 μg/mL lipopolysaccharide (LPS, Sigma-Aldrich). Then, transwell inserts containing adherent monolayers of CT26 cells were transferred to 24-well plates containing RAW246.7 cells in the lower chamber. One percent supernatant of EcN wt and the engineered strain EcN::*mcmA* (MOI = 10) were added to transwell inserts, and the cells were cultured for 5 h. At last, the mRNA expression and secretion of IL-1β, TNF-α, and IL-6 on macrophages induced by LPS were measured by RT-PCR and ELISA.

## 5. Conclusions

To sum up, we discovered that the microcins appeared not to be responsible for the inhibition of EHEC O157: H7, but microcin MccM was more closely related to the activity of EcN against *Salmonella* than expected. The growth, adhesion, and invasion of SE and ST were decreased more by EcN::*mcmA*. We developed an engineered probiotic, EcN, as the producer of overexpressing MccM to antagonize pathogenic enterobacteria, which opens the way to providing substitutes for antibiotics.

## Figures and Tables

**Table 1:** Materials including strains, plasmids, and primers used in our study.

Materials	Description	Description
**Strains**		
DH5α	Plasmid amplification host	Our lab
EcN	*E. coli* Nissle 1917, wt	Our lab
EcN Δ*mcmA*	Knockout of EcN Microcin M precursor, mutant strain	Our study
EcN Δ*mchB*	Knockout of EcN Microcin H47 precursor, mutant strain	Our study
EcN Δ*mcmA*Δ*mchB*	Knockout of EcN Microcin M and H47 precursor, mutant strain	Our study
EcN::*mcmA*	Overexpression of EcN Microcin M precursor, engineered strain	Our study
EHEC O157:H7	Enterohemorrhagic *E. coli* (EHEC) O157:H7 ATCC 35150	Our lab
SE	*Salmonella enterica* subsp. e*nterica* serovar *Pullorum str.* ATCC 9120	Our lab
ST	*Salmonella enterica* subsp. *enterica* serovar *Typhimurium* ATCC 14028	Our lab
**Plasmids**		
pUC19	Expressing GFP (green) or mCherry (red) fluorescent proteins, Overexpression vector	Our lab
pTargetF	Plasmid for CRISPR-Cas9	Our lab
pCas	Plasmid for CRISPR-Cas9	Our lab
**Primers**		
A20-F	GGATGATAATCCTATACCTGgttttagagctagaaatagca	
A20-R	CAGGTATAGGATTATCATCCactagtattatacctaggact	
B20-F	GTTAAGATATATTTCCGGGGgttttagagctagaaatagca	
B20-R	CCCCGGAAATATATCTTAACactagtattatacctaggact	
AH1-F	agagtcgacctgcagaagcttGGGGTATGAAACGTTAATAGGT	
AH1-R	taaggtTTCAACACCTTCGCTATAAGATT	
AH2-F	gcgaaggtgttgaaACCTTATATTGTTAATGAAGCACC	
AH2-R	ggagctgcacatgaactcgagTTTTATGGATTAATATATTTTCTTCTGT	
BH1-F	agagtcgacctgcagaagcttGACTTTATATGGACAATATGACACTTT	
BH1-R	aaatataaATAAACTCCATCATATTTAACTTCC	
BH2-F	gatggagtttatTTATATTTTTATTTATTTTACAGGTACTTT	
BH2-R	ggagctgcacatgaactcgagAACGTGCACCACCTCCAT	
Δ *mcmA*-F	GAAGAAAAGAAACCGGAAATT	
Δ *mcmA*-R	ACTTCTTGTTCCTGTATATGAAGAGA	
Δ *mchB*-F	GTTTGCAAAAATGTTTGTTATAGG	
Δ *mchB*-R	AACGTGCACCACCTCCAT	
pUC19-F	ATGACCATGATTACGCCAAG	
pUC19-R	AGCTGTTTCCTGTGTGAAATTG	
pUC -*mcmA*-F	ttcacacaggaaacagctATGAGAAAACTATCTGAAAATGAAAT	
pUC -*mcmA*-R	tggcgtaatcatggtcatTTAGTGGTGGTGGTGGTGGTGACTTCCACTCCCCGC	
STX1-SG-F	CATTACAGACTATTTCATCAGGAGGTA	
STX1-SG-R	TCGTTCAACAATAAGCCGTAGATTA	
STX2-SG-F	GCGGTTTTATTTGCATTAGC	
STX2-SG-R	TCCCGTCAACCTTCACTGTA	
IC-1F	GACCACTACCAGCAGAAC	
IC-10R	CTTGTACAGCTCGTCCATGC	

Lowercase letters indicate homologous arm of restriction-free cloning (RF-cloning) and enzyme digestion sites HindIII and Xhol.

## Data Availability

The raw data supporting the conclusion of this article will be made available by the authors without undue reservation.

## Supplementary Materials

The following supporting information can be downloaded at: https://www.mdpi.com/article/10.3390/ijms241411688/s1.
