# Supplementary material for: Antibacterial MccM as the Major Microcin in Escherichia coli Nissle 1917 against Pathogenic Enterobacteria
Source: Int J Mol Sci. 2023 Jul 20;24(14):11688. doi: 10.3390/ijms241411688 (PMC10380612; doi:10.3390/ijms241411688)
Supplement: Supplementary file 1 [file ijms-24-11688-s001.zip › ijms-2441789-supplementary.pdf]

Supplementary Figure S1| Growth curve of each strain.

Supplementary Figure S2| The effect of 2,2′-bipyridine or Fe^{3+} concentration on bacterial growth.

Supplementary Figure S3| Effect of EcN on *stx1* and *stx2* gene expression of EHEC O157:H7. EcN and EHEC O157:H7 co-cultured in LB medium (**a, b, d**) or medium supplemented with 4 mM iron citrate (**c**) at 2 h (**a**), 4 h (**b**), and 24 h (**c, d**). The RT-PCR data showed the fold change in expression in co-cultures (EcN or EcN mutants) relative to EHEC O157:H7 alone and normalized to an exogenous housekeeping gene control (IC-RNA) [1]. Data (means ± standard errors) were from three independent experiments. * $p < 0.05$.

Supplementary Figure S4| Spectrofluorometric assays. Wild-type EcN and SE/ST express the fluorescent proteins mCherry and eGFP, respectively. After 4 hours of co-culture in LB medium, the survival number of bacteria was characterized by fluorescence number.

Figure S1

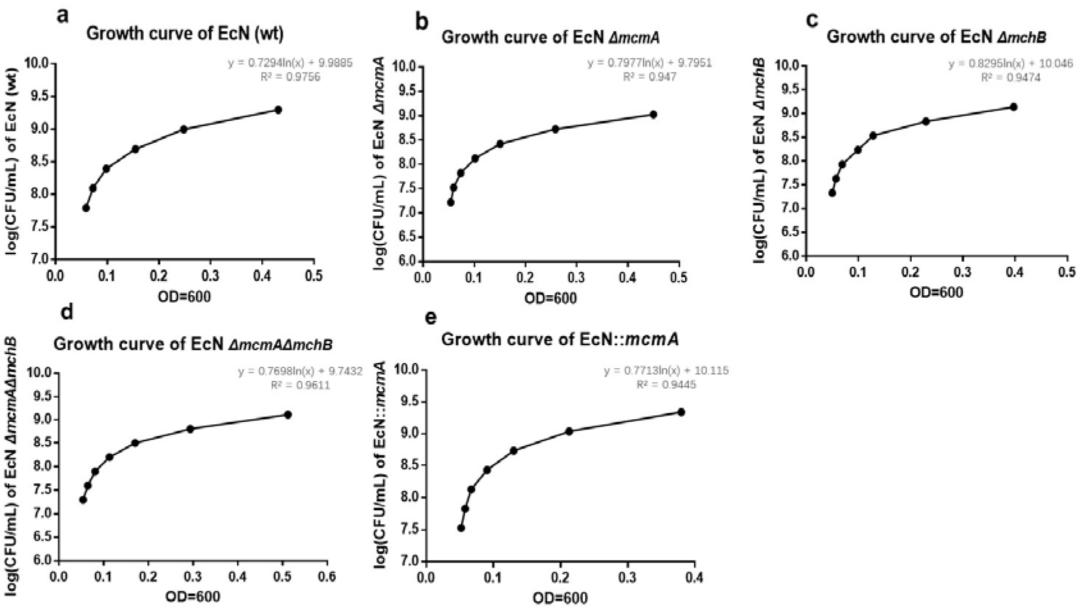

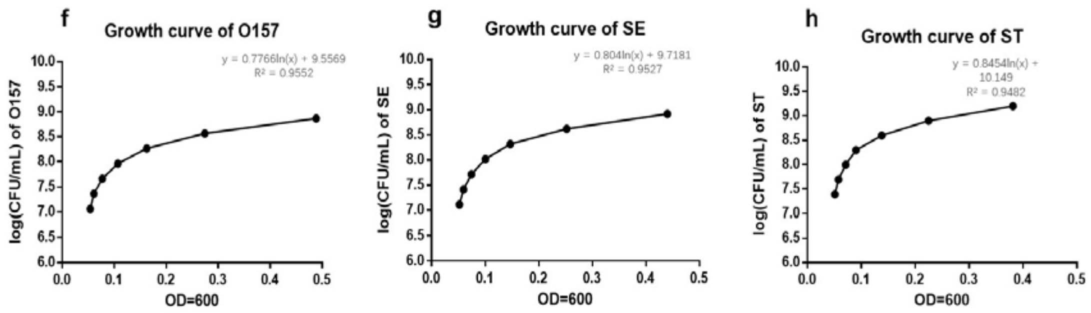

Figure S2

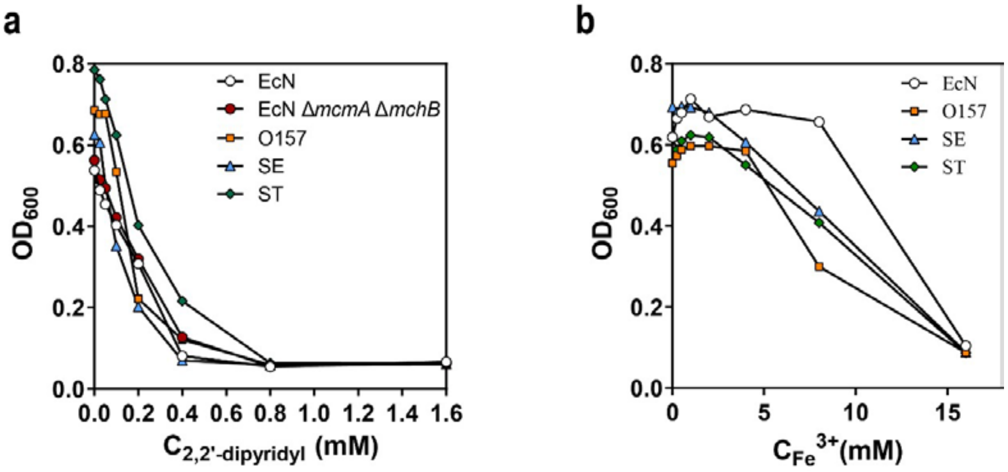

Figure S3

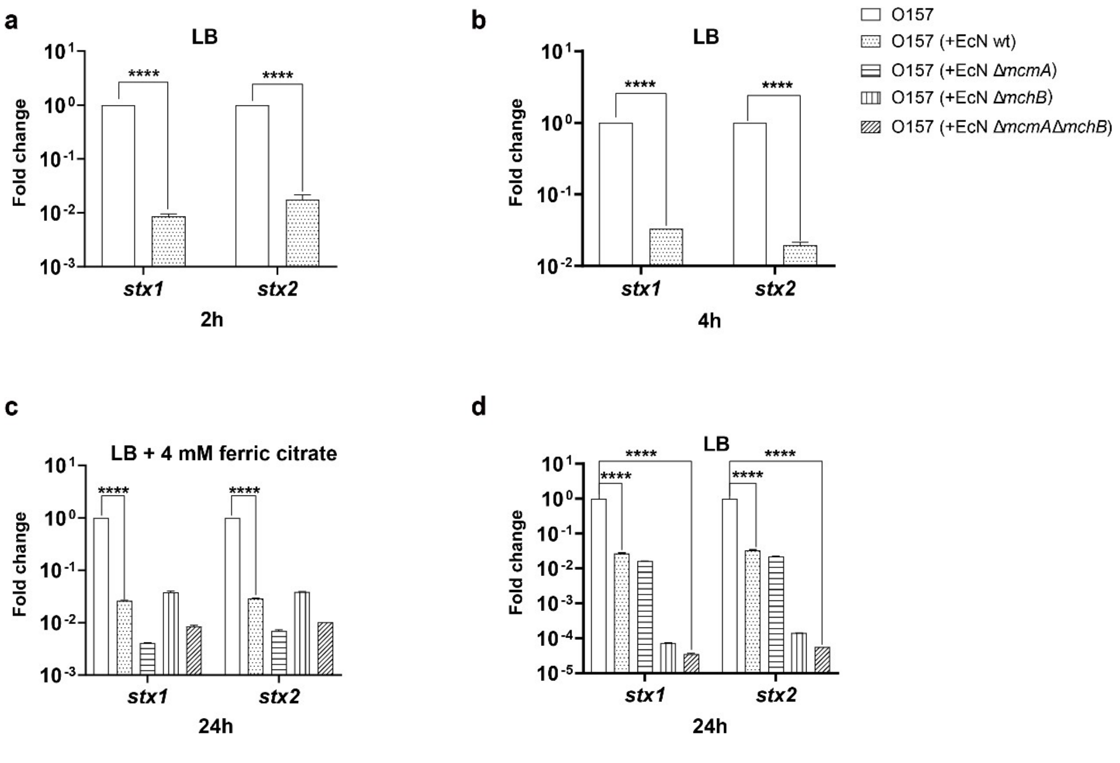

Figure S4

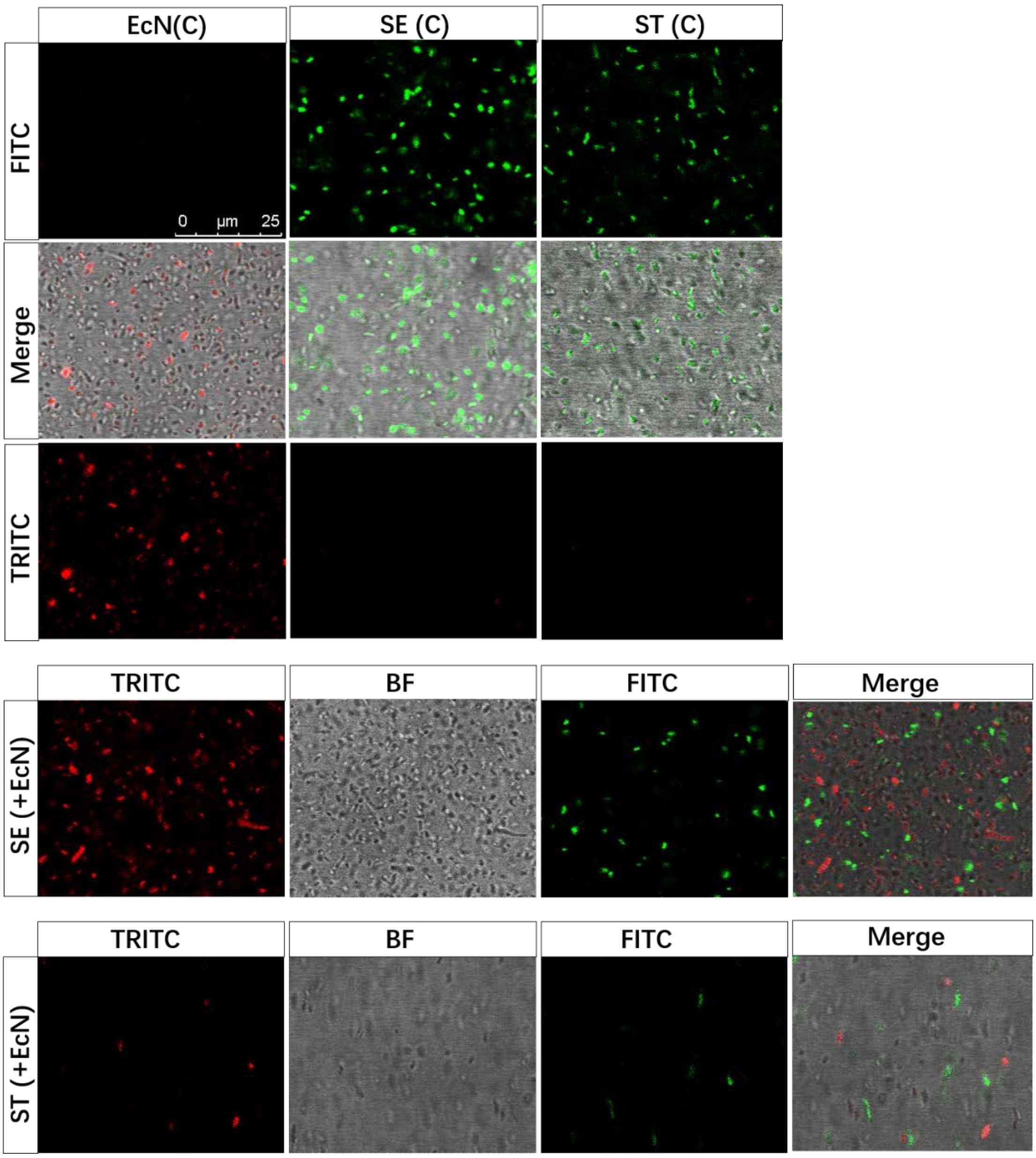

References

1. Mohsin, M.; Guenther, S.; Schierack, P.; Tedin, K.; Wieler, L. H., Probiotic Escherichia coli Nissle 1917 reduces growth, Shiga toxin expression, release and thus cytotoxicity of enterohemorrhagic Escherichia coli. Int. J. Med. Microbiol. **2015,** 305, (1), 20-26.
